# Collisional formation of top-shaped asteroids and implications for the origins of Ryugu and Bennu

**DOI:** 10.1038/s41467-020-16433-z

**Published:** 2020-05-27

**Authors:** P. Michel, R.-L. Ballouz, O. S. Barnouin, M. Jutzi, K. J. Walsh, B. H. May, C. Manzoni, D. C. Richardson, S. R. Schwartz, S. Sugita, S. Watanabe, H. Miyamoto, M. Hirabayashi, W. F. Bottke, H. C. Connolly, M. Yoshikawa, D. S. Lauretta

**Affiliations:** 1 Universite Côte d’Azur, Observatoire de la Côte d’Azur, Centre National de la Recherche Scientifique, Laboratoire Lagrange, Nice, France; 2 Lunar and Planetary Laboratory, University of Arizona, Tucson, AZ USA; 3 The Johns Hopkins University Applied Physics Laboratory, Laurel, MD USA; 4 Physics Institute, University of Bern, NCCR PlanetS, Gesellsschaftsstrasse 6, 3012, Bern, Switzerland; 5 Southwest Research Institute, Boulder, CO USA; 6 London Stereoscopic Company, London, UK; 7 Department of Astronomy, University of Maryland, College Park, MD USA; 8 Department of Earth and Planetary Science, School of Science, The University of Tokyo, Tokyo, Japan; 9 Graduate School of Environmental Studies, Nagoya University, Nagoya, Japan; 10 Department of System Innovation, School of Engineering, The University of Tokyo, Tokyo, Japan; 11 Department of Aerospace Engineering, Auburn University, Auburn, AL USA; 12 Department of Geology, School of Earth and Environment, Rowan University, Glassboro, NJ USA; 13 Institute of Space and Astronautical Sciences, JAXA, Sagamihara, Japan

**Keywords:** Astronomy and planetary science, Planetary science, Asteroids, comets and Kuiper belt

## Abstract

Asteroid shapes and hydration levels can serve as tracers of their history and origin. For instance, the asteroids (162173) Ryugu and (101955) Bennu have an oblate spheroidal shape with a pronounced equator, but contain different surface hydration levels. Here we show, through numerical simulations of large asteroid disruptions, that oblate spheroids, some of which have a pronounced equator defining a spinning top shape, can form directly through gravitational reaccumulation. We further show that rubble piles formed in a single disruption can have similar porosities but variable degrees of hydration. The direct formation of top shapes from single disruption alone can explain the relatively old crater-retention ages of the equatorial features of Ryugu and Bennu. Two separate parent-body disruptions are not necessarily required to explain their different hydration levels.

## Abstract (web summary)

Asteroid shapes and hydration levels can serve as tracers of their history and origin. Here, the authors show top shape asteroids can form directly through gravitational reaccumulation and rubble piles formed in a single disruption can have similar porosities but variable degrees of hydration.

## Introduction

Images of the low-albedo near-Earth asteroids Ryugu and Bennu, respectively taken by the JAXA’s Hayabusa2 and NASA’s origins, spectral interpretation, resource identification, and security–regolith explorer (OSIRIS-REx) sample-return space missions, show that these two small asteroids have spinning top-like shapes^1,2^. Such asteroids have an oblate spheroidal shape with a more or less pronounced equatorial feature that, for simplicity, we call a ridge hereafter. Spin-up via the Yarkovsky–O’Keefe–Radzievskii–Paddack (YORP) effect^3^ is frequently attributed as the main factor for formation of asteroids with top shapes^4^. However, the equatorial ridges of both Ryugu and Bennu appear to be their oldest surface features, predating the formation of large equatorial craters^1,5,6^. These geologic characteristics suggest that Ryugu and Bennu formed directly as top-shapes, or achieved such a shape early after their formation. Spectral observations of their surfaces reveal a hydration feature that is deeper on Bennu than on Ryugu^6–8^. These observations suggest that the two bodies have different levels of hydration, assuming that the mineralogy of their surfaces is representative of their bulk compositions.

The collisional lifetime of asteroids of the sizes of Ryugu and Bennu (equatorial radii of 502^1^ and 253 m^9^, respectively) is short compared to the age of the Solar System^10^. Therefore, these asteroids are likely fragments of larger bodies that were disrupted by a collision with other bodies in the asteroid belt^11–13^. Such events could have left a trace in the form of asteroid families^14^. Ryugu and Bennu were transported to near-Earth space through well-identified dynamical routes^15,16^. A fundamental question to understand the origin and history of top-shape low-albedo asteroids, like Ryugu and Bennu, is whether their bulk shape and hydration level could be the immediate consequence of their formation following the disruption of their parent body. Answering this question should lead to new paths toward understanding asteroid shapes and hydration levels and provide insights into the relationship between these characteristics, candidate parent bodies, and the specifics of the disruption event in the asteroid belt.

Here, we perform a series of simulations of disruptions of 100-km-diameter asteroids with microporosity, as expected for parent bodies of dark (geometric albedo < 0.1) asteroid families^17^, over a wide range of impact energies and angles. We simulate both the fragmentation and the gravitational phases of the disruption during which fragments reaccumulate and form rubble piles^18^. The resulting fragment size and ejection velocity distributions have been published^19^. We then compute the gravitational phase using the approach of ref. ^20^ to track the shapes of aggregates formed by reaccumulation and compare these shapes with those of Ryugu and Bennu. We also track the peak temperature experienced by the components of each aggregate as a result of the asteroid parent body disruption to determine the hydration state of different aggregates formed in a single disruption from the same starting material. We then show that oblate spheroids are commonly formed as reaccumulated rubble piles, leading in some cases to spinning top shapes. Moreover, rubble piles like Bennu and Ryugu with similar porosities but variable degrees of hydration can form in a single disruption.

## Results

### Diversity of rubble-pile shapes

**Fig. 1:**
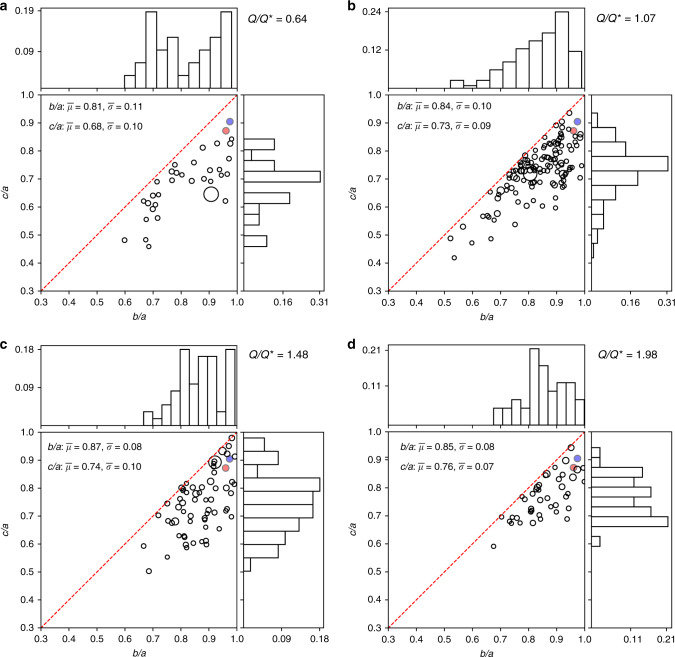
Reaccumulated aggregates can have a wide range of shapes. For four different conditions that range in impact energy (represented by the impact energy relative to the calculated catastrophic disruption threshold, *Q*/Q*), we show the axial ratios of the reaccumulated remnants, represented by a single point in each plot. From **a** to **d**, the impact energy *Q* is increased from below Q* (**a**) to above Q* (**b**–**d**). The marginal distributions of the minor-to-major axis ratios (*c*/*a*) and the intermediate-to-major axis ratios (*b*/*a*) are also presented. The relative radius of each aggregate is represented by the radius of its data point. The axial ratios of Ryugu and Bennu are shown as blue and red points, respectively (Note: the sizes of these points do not reflect the relative size of Ryugu and Bennu to the aggregates formed in these simulations. The maximum resolution attained in our simulations results in aggregates that are larger by a factor of 4–8 than Ryugu and Bennu). The mean, $\bar \mu$, and standard deviation, $\bar \sigma$, of the axis ratios of all reaccumulated aggregates in each run are shown in the legends of the panels. For these impact conditions, we find that the higher the impact energy, the more spherical the average aggregate.

We used the output of the fragmentation phase of the asteroid disruptions modeled by Jutzi et al.^19^ to perform four gravitational-phase simulations (Table 1), using the soft-sphere discrete element method (SSDEM)^21^ (see Section “Improvements in gravitational phase modeling”) and assuming specific sets of values of friction parameters to compute the contact forces between the particles reaccumulating to form aggregates. Figure 1 shows the final shapes of all aggregates with more than 15 individual particles in four simulations covering a representative range of impact conditions and assuming friction coefficients commensurate with a global aggregate angle of friction of 18° (see Section “SSDEM simulation parameters”), which is consistent with the angle of friction estimated for Bennu^9^. We performed simulations at different angles of friction and find that increasing the angle of friction tends to lead to a larger fraction of aggregates having more prolate shapes (Supplementary Fig. 1).

**Table 1:** Summary of simulation parameters and outcomes.

case	*θ*_imp_ (°)	*R*_imp_ (km)	*Q*/Q*	*F*_oblate_	Δ*T*_center_ (K)	*C*_center_
1	30	7	0.635	0.22	+45.9	0.64
2	15	7	1.066	0.41	+104.3	0.91
3	30	9	1.481	0.48	+113.0	0.93
4	30	13	1.979	0.65	+247.7	1.00

We summarize the impact parameters of the four simulations that have a similar impact speed of 5 km/s and variable impact angle (*θ*_imp_) and impactor diameter (*R*_imp_). We report the corresponding impact energy relative to the catastrophic disruption threshold (*Q*/Q*, see ref. ^62^), the fraction of reaccumulated aggregates that are oblate spheroids (arbitrarily defined as having a minor-to-major axis ratio > 0.75, *F*_oblate_), the impact-induced change in temperature at the center (Δ*T*_center_) and the degree of compaction at the center (*C*_center_). An object is fully compacted (no microporosity) when *C* = 1 and has its original microporosity when *C* = 0. The center is defined as the subset of material originally within a radius of 5 km from the center of mass of the parent body.

**Fig. 2:**
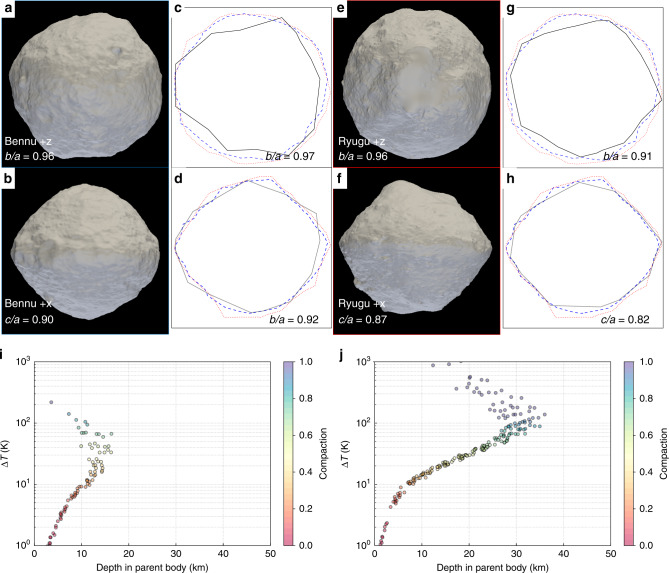
Reaccumulated aggregates can resemble Bennu and Ryugu. **a**–**h** Comparison of the Bennu (**a**, **b**) and Ryugu (**e**, **f**) shape models^1,9^ and the shape profiles of two reaccumulated aggregates (**c**, **d**, **g**, **h**) from simulation 3 (Table 1). The panels **a**, **c**, **e**, **g** show the profile of each shape from a polar view, and the panels (**b**, **d**, **f**, **h**) show the profiles from an equatorial view. The shapes of the reaccumulated remnants are traced in black and are compared to the traced shapes of Bennu (blue dashed line) and Ryugu (red dotted line). **i** The temperature change, Δ*T* (*y-*axis), the original depth in the parent body (*x*-axis), and the degree of compaction (color, unitless) of each particle that makes up the simulated aggregate shown in (**c**) and (**d**). **j** The same properties as in **i** are shown for the simulated aggregate shown in (**g**) and (**h**). **i**, **j** The particles that experience the most heating and compaction originate near the surface, close to the impact point. Those experiencing the least heating and compaction also originate near the surface, but closer to the antipode of the impact. Although the two reaccumulated remnants (shown in **c**, **d**, **g**, **h**) have very similar shapes, they apparently have different thermal histories. Comparing **i** and **j**, we find a clear difference in the peak and average temperature change and the degree of compaction experienced by the material that formed these two aggregates. Furthermore, the aggregate shown in **g** and **h** is composed of material that is sampled from a larger maximal depth within the parent body.

The final shapes of aggregates in our simulations span a wide range of aspect ratios, and we observe an increase in the fraction of oblate spheroids, like Ryugu and Bennu, for higher specific impact energies. Such an oblate spheroidal shape for a rubble pile is a necessary starting condition to lead to top-shaped asteroid formation by YORP spin-up, given plausible assumptions about the internal structure of the aggregate^22,23^. Here, we show that appropriate starting conditions are actually a natural and common outcome of the reaccumulation process that drives rubble pile formation. Moreover, by analyzing in detail the shapes of oblate aggregates after the reaccumulation process (Fig. 2), we find that they can have top shapes similar to those of Ryugu and Bennu. However, our simulations are still too coarse to resolve the topographic details.

After the formation of an oblate spheroid, it should not take much time (order of 10^4^–10^6^ years for kilometer-sized asteroids^3,24^) for post-processes such as YORP to lead to top shapes such as those currently observed. Dynamical studies of the spins of main belt asteroids show that YORP evolution is required to explain their distribution^25^. Measurements of Bennu’s YORP acceleration show that its spin-rate doubling period is roughly 1.5 Myr at its current orbit^26^. In the inner main belt, this timescale would be ~6 Myr. Observations of Ryugu and Bennu require a model that forms a top shape rapidly such that subsequent impact cratering can form large craters that overlay the equatorial ridge^5,27^. The YORP timescale provides ample time for the transformation of an oblate spheroid to a top shape before the formation of the largest craters. For example, Bennu’s largest equatorial craters (~100 m diameter) may require 0.1–1 Gyr residence time in the main belt to form^5^. In that scenario, large-scale YORP deformation following gravitational reaccumulation would be plausible.

This scenario also implies that any subsequent action by YORP would not lead to further global shape deformations. Our simulations which lead to a rapid formation of top shapes like Ryugu and Bennu thus provide a solution to the preservation of large craters on the equatorial ridges of bodies with these shapes. In addition, Bennu and Ryugu have been linked to asteroid families in the inner main belt that have dynamical ages of ~1 Gyr^12^. If the ridges on those bodies formed at approximately the same time, they would be subject to a billion years of impacts from main belt projectiles. The survival of the ridges therefore depends on the nature of the crater scaling rule translating projectile sizes into large crater sizes. If pure gravity scaling is assumed, as suggested by the Small Carry-on Impactor experiment performed by Hayabusa2 on Ryugu, our expectation is that the ridges would have been blasted away over that timescale. For Bennu and perhaps Ryugu, however, there is evidence that large craters could be interacting with stiffer interior^9^. This could indicate that crater scaling for large craters must include strength effects, which in turn would allow the ridges to survive the long transit of Bennu and Ryugu to their current orbits.

### Dynamical paths to top-shaped asteroids through gravitational reaccumulation

The YORP effect is generated by thermal pressures caused by the reflection and re-emittance of light from the surface of an asteroid. These torques can modify an asteroid’s spin axis, with obliquity approaching either 0° or 180°, and can lead to asteroid spin-up—that is, an increase in rotation rate^28^. Spin-up can be sufficient to trigger surface mass movements, depending on structural conditions, as well as the generation of a satellite, thereby potentially leading to the top shape of some asteroids and the formation of binary systems^22,29,30^, and even complete disruption of a rubble pile^31^. However, observations that asteroids have the expected obliquity and are top-shaped may not be sufficient to confirm the hypothesis that they were shaped by the YORP effect. It has been showed that time scales needed to reorient an asteroid that is already symmetric in shape are much shorter than that of YORP spin-up^24^. In addition, the YORP spin-up process is not linear, as small changes in the surface topography driven by asteroids’ rotation can substantially vary the YORP acceleration^32^. Therefore, although the YORP effect has been measured for near-Earth objects^26,33^, it is still unclear how it directly leads to top shapes.

**Fig. 3:**
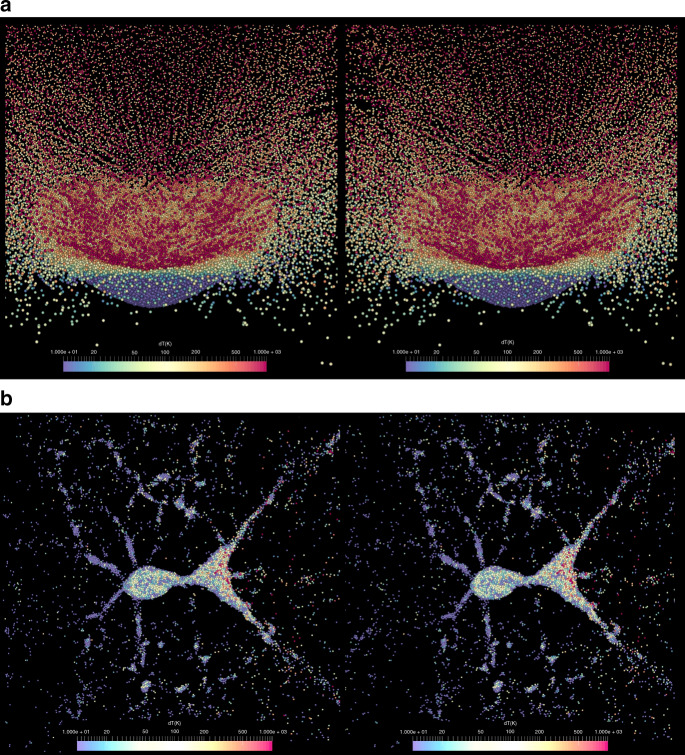
A catastrophic disruption can be at the origin of thermal alteration. **a** Stereoscopic pair at time *t* = 0 of the gravitational phase of the disruption of the parent body for simulation 3 (see Supplementary Movie 1). The peak temperature change for each particle from the impact is shown (see also Fig. 5) with colors scaling from 10 K (blue) to 1000 K (red). **b** Stereoscopic pair of the gravitational reaccumulation of the largest remnant (see Supplementary Movie 2) at *t* = 4.75 hours, exhibiting filamentary structure that will eventually collapse into a single aggregate. Other smaller filaments will escape and collapse to form smaller aggregates. This stereoscopic pairs in the Supplementary Movies can be viewed in ‘parallel’ stereo mode simply by relaxing the eye convergence. We suggest to view the pair of images from a foot or so away, and look through the screen to infinity, allowing the two images to float across each other. Where the two central pictures exactly overlap, the ‘fused’ 3-D image is to be found; all that is then necessary is to gently adjust the focus of the eyes, while the convergence remains relaxed, to obtain a clear stereoscopic image. This technique is called ‘Free Viewing’ of stereo pairs. For a more authentic stereo effect, use a Brewster format stereoscope — The London Stereoscopic Company OWL or similar. Detailed instructions may be found at LondonStereo.com.

**Fig. 4:**
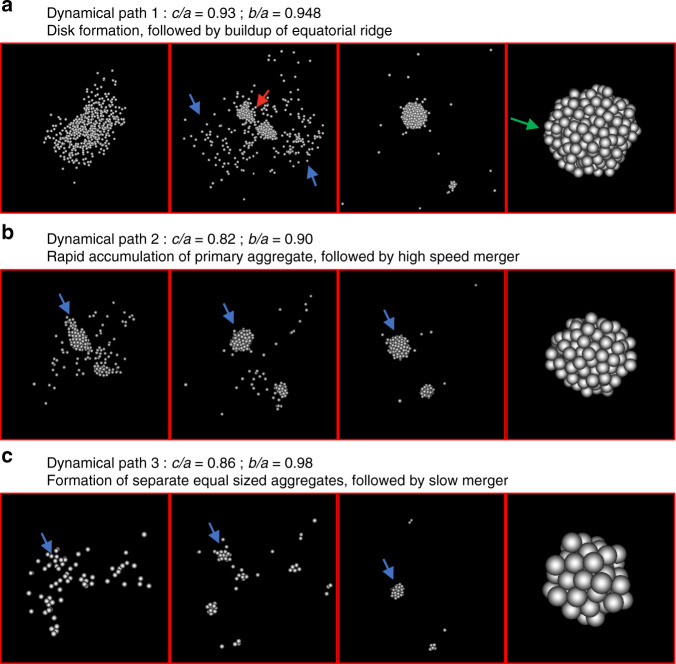
Multiple dynamical paths lead to the formation of oblate spheroids. Snapshots of three cases of the gravitational reaccumulation of those smaller aggregates are in panels (**a**–**c**). Simulations snapshots at time steps 1 min, 0.75 h, 2 h, and 5 h after the collision, separated by red lines, are given for each dynamical path presented in panels (**a**–**c**). The first panel of each case shows the immediate distribution of the particle that reaccrete to form the final aggregate shown in the last panel of the same case. **a** Path 1 shows the ejection of a material stream that forms an elongated disk (blue arrows) with a dense core (red arrow). The disk of material then accretes onto the equator, spinning up the core through conservation of angular momentum and building up a ridge (green arrow). Paths 2 and 3 (**b**, **c**) show the formation of oblate spheroids through the collapse of multiple cores that subsequently coalesce. **b** For path 2, the aggregates have a high enough relative speed that the smaller bodies break up on impact and individual particles accrete onto the larger body (blue arrow) isotropically. **c** For path 3, a kind of nucleation occurs, where a large primary aggregate quickly forms out of the merger (blue arrow) and becomes the focus point of a gradual deposition of smaller aggregates that accrete isotropically, slowly building up an oblate spheroid (see Supplementary Movies 3–5 of these three paths).

Shape distributions of the smallest rubble-pile remnants following catastrophic disruption have not been explored in detail before. Our findings show that a large fraction of reaccumulated remnants provide the correct shape or initial conditions to form top-shapes, providing a necessary alternative to YORP spin-up as the sole mechanism. Moreover, our simulations reveal dynamical paths to top shapes during the gravitational phase of a disruption while the material is ejected and reaccumulates (Fig. 3). We identified three basic dynamical paths that lead to the formation of top-shape asteroids (Fig. 4).

Overall, we find that the formation of top shapes is a complex interplay among the relative speeds of the ejected material that forms the transient aggregates that eventually coalesce. The final shapes are also determined by spatial distribution (whether the components are ejected as an elongated stream or as a spherical cloud) and angular momentum. In some circumstances, when the relative speeds between these transient aggregates are low, they collide and keep their shape, resulting in an elongated or bilobate shape (Supplementary Fig. 2). Similarly, if aggregates have high strength (higher static friction), they keep their shape at impact.

We only find ridge formation in one of the paths (path 1). In the case of the other two, the direct formation of a ridge does not occur; however, the formation of an oblate spheroid sets the appropriate initial shape for the rapid formation of a ridge through a mechanism that can rotationally accelerate the asteroid.

### Impact-driven hydration diversity in rubble piles

We computed the maximum (peak) temperature change and compaction that every particle experienced during the fragmentation phase of the disruption simulations considered here. Compaction measures the increase in the density of the individual particle relative to its initial uncompacted state. We transfer these thermodynamic properties during the handoff to the SSDEM simulations, so that each particle has a record of the peak temperature change and compaction experienced during the fragmentation phase. Ignoring the potential effects of the kinetics of the material heating over time^34^^,^ we study the instantaneous effect of shock heating. Hence, we determine the impact-heating and compaction history of each aggregate formed during the gravitational reaccumulation phase.

**Fig. 5:**
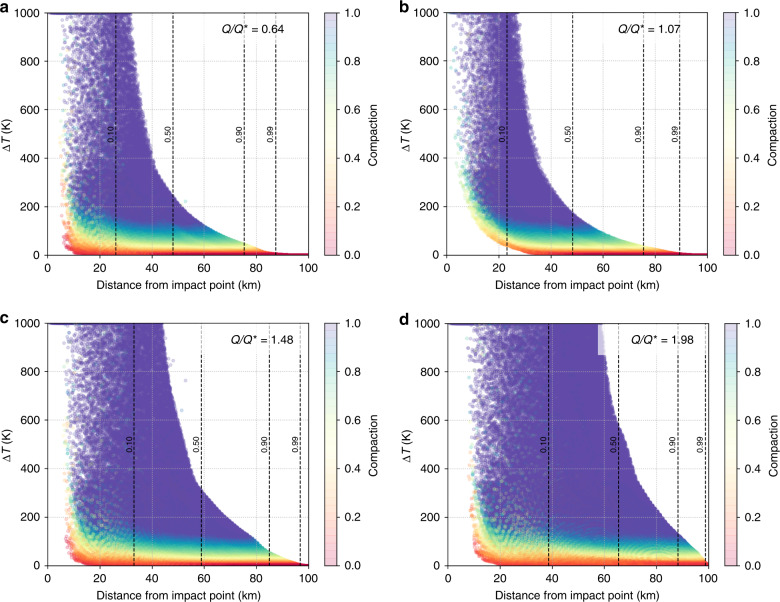
Super-catastrophic disruptions produce a large diversity of thermally altered rubble-piles. From **a** to **d**, the impact energy *Q* is increased from below the impact energy threshold for disruption Q* (**a**) to above Q* (**b**–**d**). For each simulation, we show the peak temperature change of each particle (*y-*axis), its distance from the impact point (*x-*axis), and its postimpact compaction (color scale). Particles closer to the impact point are more heated and compacted, and particles that experience a change in temperature of $\gtrsim$200 K are almost fully compacted. The vertical black dashed lines highlight different fractions of escaping material that originates from within a given distance of the impact point. For increasingly higher impact energies, escaping material originates at further distances from the impact point (for example, **c** shows an impact where *Q*/Q* = 1.481, which results in 50% of the escaping material originating from >60 km away from the impact point). Three-dimensional visualizations of the peak temperature changes and compaction of material in the parent body are presented in Supplementary Figs. 3 and 4.

We find that particles that experience a change in temperature of $\gtrsim$200 K are almost fully compacted (Fig. 5). However, the impact energies leading to this heating may be sufficiently high to cause subsequent effects, such as the sublimation of gases that may create microfractures that open up void spaces in the matrix material ^35^. This would compensate for impact-induced compaction by increasing the microporosity of the material. Therefore, it is difficult to ascertain what the true final microporosity of this material might be. However, this diversity in compaction levels of ejected material may explain the diversity found in the meteoritic record^17^.

**Fig. 6:**
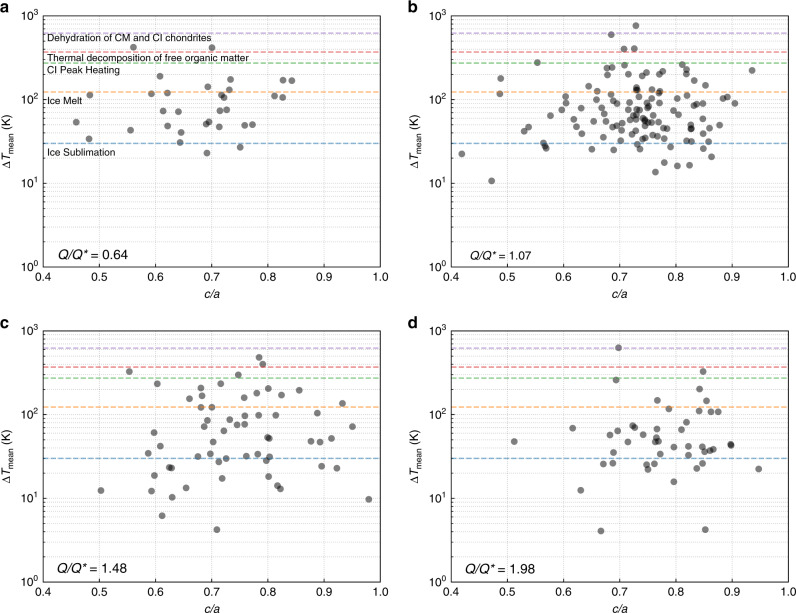
A catastrophic impact thermally alters material that forms rubble-pile asteroids. From **a** to **d**, the impact energy *Q* is increased from below the impact energy threshold for disruption Q* (**a**) to above Q* (**b**–**d**). For every reaccumulated aggregate in a simulation, we calculate the mean change in temperature by averaging the peak temperature changes of each component particle. We plot this value as a function of the minor-to-major axis ratio (*c*/*a*), which tracks the sphericity of each represented object. We find that the aggregates are able to experience mean temperature changes that likely altered the material from its original state within the parent body. The degree of alteration is independent of the final asteroid shapes, and different spheroidal or top-shaped asteroids can have varying degrees of thermal alteration driven by impact-induced heating (as shown for the two aggregates in Fig. 2). Unintuitively, rubble piles generated from less energetic impacts are more likely to be thermally altered, and super-catastrophic impacts produce a more diverse population of objects. Assuming an initial isothermal target with a temperature of 150 K^59^, we highlight the change in temperature thresholds for ice sublimation (180 K, blue dashed line), ice melt (273 K, orange dashed line), CI peak heating (423 K, green dashed line^60^), the thermal decomposition of free organic matter (520 K, red dashed line^61^), and the dehydration of CM and CI chondrites (773 K, purple line^36^).

We generally find that for a given aggregate shape, the average peak temperature change of the particles that compose that aggregate can cover a wide range (Fig. 6). Specifically, the average extends well above and below the threshold temperature at which minerals start to be dehydroxylated during impact-heating at 500 °C^36^. Therefore, we can expect aggregates of the same shape to have a different level of hydration. Thus, the different level of hydration observed for Ryugu and Bennu do not necessarily mean that they come from two different parent bodies having two distinct thermal histories, or that they experienced different surface heating histories after formation due to solar radiation^16,37^. Rather, it could be the outcome of the disruption of a common parent body, regardless of its internal heating history^6^. Figure 2b shows that two aggregates with top-like shapes can sample different parts of the parent body that experienced different heating at impact. These cases illustrate larger reaccumulated aggregates that are made of material originating from different distances from the impact point on the parent body. Therefore, each of these aggregates contains a mixture of material with different hydration levels, and each can also have a different global hydration level.

In contrast, the smaller rubble piles are sourced from localized regions; therefore, our simulations predict that Ryugu and Bennu, as likely some of the smallest members of their asteroid family, would each be created with a homogeneous hydration level, though differing between the two bodies. This is consistent with the uniform degree of hydration suggested by the spectral observations^7,8^.

This conclusion is predicated on the difference in the observed band depth of the 2.7-micron feature on the surface of both bodies. It may also be that the interiors of Ryugu and Bennu are equally hydrated, and that the difference comes from post-formation processes. Even in this case, they could still originate from the same disruption, as our simulations show a wide range of impact-induced temperature changes in escaping material, meaning that multiple reaccumulated aggregates can have similar global hydration levels. Analysis of the returned samples from the Hayabusa2 mission, which obtained material from both the surface of Ryugu and possibly also its near-subsurface, and OSIRIS-REx, which will return samples from the surface of Bennu, will shed light on this issue.

The results of our simulations have important consequences for the identification of asteroid families of low-albedo bodies based on the observed presence of a 0.7 or 2.7-micron hydration feature in their spectrum. Considering Ryugu and Bennu, dynamical models suggest that they originate from the inner part of the asteroid belt^38,39^. There are several families in the inner belt that could be related to these bodies^38,40^. In particular, four possible source families in the inner main belt have been identified: Polana–Eulalia, Clarissa, Erigone, and Sulamitis. Whereas most members in the Polana–Eulalia complex and Clarissa families have no hydration band identified in the visible spectral range, the majority of the members in the Erigone and Sulamitis families do^40^. Our results indicate that the presence of a hydration band itself is not diagnostic of membership within a family; however, our results indicate a correlation between the impact energy level and family member homogeneity. In particular, we predict that for higher specific impact energies, the abundance of hydrated fragments is lower. Thus, the creation of these two classes of dark-type families, based on the abundance of members with the 0.7-micron feature, must have occurred at very different specific impact energies.

## Discussion

The number of small km-size bodies that form from the catastrophic disruption of a 100-km size parent body is quite large, on the order of ~10^4^-10^5^ individual asteroids^19^. After their formation, roughly half end up drifting inwards to a resonance that would inject them into Near-Earth space^15^. Therefore, Bennu and Ryugu could be twin rubble piles that experienced this dynamical path.

However, it was argued that Ryugu’s partial dehydration could not be explained by a single impact that disrupted its original parent body^6^; rather, Ryugu was likely formed in a sequence of catastrophic disruptions, starting from an internally heated parent body (Fig. 8 of ref. ^6^). In light of comparable observations at Bennu, we provide a single-model-solution that may explain the characteristics of Ryugu and Bennu. In particular, these asteroids have very similar bulk densities (1.19 g/cm^3^;^1,9^), but exhibit different depths in their hydration band^7,8^. In the context of a single impact origin, these characteristics require a formation model that can result in these commonalities and differences.

**Fig. 7:**
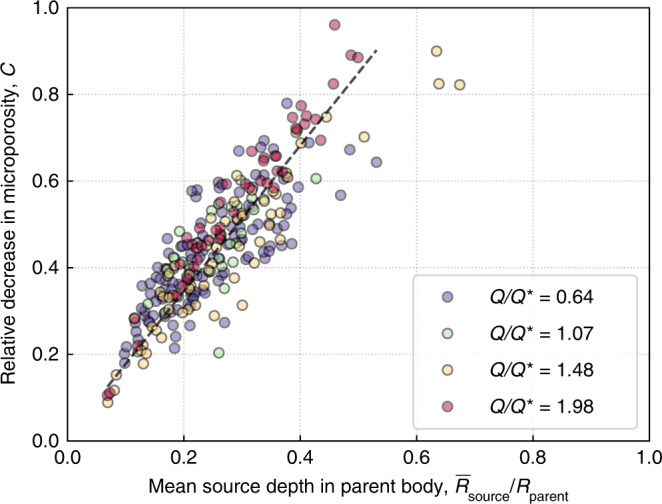
Microporosity can be a predictor of the material provenance within the parent body. The mean compaction of the particles that make up an individual rubble-pile aggregate in our simulations is a measure of the microporosity of that aggregate. We show the mean compaction for all four cases described in Table 1. The color of each scatter point represents its associated simulation: purple, green, yellow, and red circles are aggregates from the *Q*/Q* = 0.64, 1.07, 1.48, and 1.98 cases, respectively. We find that the change in a rubble pile’s microporosity relative to that of the parent body material (*y*-axis) is an indicator of that material’s original location from within a parent body (*x-*axis, parameterized by the mean source region of all particles making up the rubble pile, $\bar R_{{\mathrm{source}}}$, normalized by the parent body radius, $R_{{\mathrm{parent}}}$), regardless of the energy of the family forming impact. This is illustrated by the black dashed line, which is a linear fit to data from all simulations, and has the form *C* = (1.68 ± 0.02) $\bar R_{{\mathrm{source}}}/R_{{\mathrm{parent}}}$.

Here, our detailed simulations show that a single catastrophic disruption of a hydrated parent body results in a family of asteroids with a high diversity of hydration and compaction levels, especially when the disruption occurs at a high *Q*/Q*. Our results suggest that there are two scenarios from a single disruption that may explain the similar bulk density (and thus the similarly porous nature) of Ryugu and Bennu, which are directly related to the region in the parent body from which they are sourced (Fig. 7).

In the first scenario, the material of the two asteroids would experience different levels of both heating and compaction at parent-body impact. They would thus have different macroporosity to achieve the same bulk density. This difference could come from how the reaccumulating aggregates’ components are interlocked. In this case, Bennu’s material would be sourced from closer to the parent body’s surface, whereas Ryugu’s would originate from nearer to its center.

In the second scenario, the two asteroids would have undergone a different level of heating but a similar level of compaction during the disruption. This scenario points to Bennu being sourced from the antipodal region, whereas Ryugu would have been sourced from near the impact point, where material can experience high heating but little compaction. In that way, they could both have the same microporosity but a different level of hydration. This scenario may also imply that the origin of the asteroid family forming these asteroids was a high-energy impact event, exceeding the catastrophic disruption threshold, so that antipodal material could escape.

In the context of a single-parent-body origin, the measured porosities in the samples returned by Hayabusa2 and OSIRIS-REx will allow us to discriminate between these two scenarios. In combination with our results, the sample analysis will provide crucial clues regarding the influence of the impact process on the observed diversity in hydration levels of carbonaceous asteroids and meteorites. Furthermore, the apparent presence of exogenous material contaminating Bennu and Ryugu, with different compositions from one object to the other^41,42^, may also help in revealing the true relationship between the two objects.

## Methods

### Modeling the fragmentation phase

Numerical simulations of asteroid disruptions, including both the fragmentation phase during which the asteroid is broken up into small pieces and the gravitational phase during which fragments may reaccumulate due to their mutual attractions and form rubble piles, were first conducted in the early 2000 s and successfully reproduced the size distributions of asteroid families^18^. The simulations, which used a smooth particle hydrodynamics (SPH) hydrocode including a model of brittle failure^43^ to compute the fragmentation phase and the parallel N-body code pkdgrav^44^ to compute the gravitational phase, showed that all fragments larger than typically 200 m are rubble piles formed by reaccumulation of smaller pieces. Other simulations using a porous material model in the SPH simulations to simulate the disruption of microporous asteroids^45^ showed the same role of the reaccumulation phase in systematically forming rubble piles for bodies larger than 200 m^46^. The formation of both Bennu and Ryugu by such a process is consistent with their low bulk density and the interpretation from Hayabusa2 and OSIRIS-REx data that they are rubble piles^1,2,6,47^.

### Improvements in modeling material impact heating and compaction

The shock physics code calculations of the collisions were performed in ref. ^19^ using the relatively simple Tillotson equation of state, which does not allow for a direct computation of a thermodynamically consistent temperature. However, we can still approximately compute the temperature. Such a computation was improved^48^ by analyzing the increase in specific internal energy to model the change in temperature caused by an impact. As shown in ref. ^49^, this approach leads to temperatures comparable to the ones computed via the more sophisticated ANEOS equation of state, as long as vaporization is negligible.

More general results are presented in ref. ^48^; however, for the sake of completeness, we describe briefly here the methodology. We used a temperature dependent heat capacity to compute the temperature increase for rocky materials. We then take into account the temperature dependence of the heat capacity through a linear relationship that approximates laboratory measurements of the heat capacity of fosterite^50^.

In order to compute the degree of compaction, *C*, caused by the collision, we consider the relative change of porosity, defined as

1 $$\begin{array}{*{20}{c}} {C = \frac{{p_0 - p}}{{p_0}} = \frac{{\alpha _0 - \alpha }}{{\alpha \left( {\alpha _0 - 1} \right)}},} \end{array}$$

where *P*_0_ = 1−1/*α*_0_ is the initial porosity, *P* = 1−1/*α* is the postimpact porosity, and *α*_0_ and *α* are the initial and postimpact distention, respectively. For full compaction, *C* = 1 while in the case of no compaction, *C* = 0.

### Modeling the gravitational reaccumulation phase

Early simulations of the reaccumulation phase of a disruption were not able to track the shapes of reaccumulated rubble piles as growing bodies were replaced by a single sphere^18,51^. Improvements in the modeling allowed assessing shapes by including a rigid aggregate model in pkdgrav^52^. In this model, reaccumulating particles can either stick at contact or bounce with assigned coefficient of restitutions, using hard sphere collisions (hard sphere discrete element method). Growing aggregates can also break into individual particles or smaller aggregate structures, depending on their assigned strength, their reimpact conditions and their experienced tidal torques. Using this method, the first resulting simulations reproduced successfully the shape of the asteroid Itokawa, as well as the presence of boulders on its surface^53^.

Our study does not capture two considerations that may contribute second-order effects that influence the final shape of a reaccumulated remnant. The first is our simplified assumption of a monodisperse size distribution. Work by refs. ^23,30,54^ have shown that a nonuniform size distribution can influence the effective internal angle of friction of a rubble pile. Those authors investigated the influence of a size distribution on the critical spin period of a rubble pile before rotational disruption; nevertheless, we surmise that this could play a role in the reaccumulation process. The second simplifying assumption in our simulations is the use of spherical particles. Recent work^56,57^ has shown that the gravitational collapse of nonspherical fragments may influence the gravitational reaccumulation process, leading to varied final shapes.

However, our initial conditions start from the actual dynamical configuration of fragments produced by the impact phase, instead of a global collapse of a cloud of fragments, as considered in refs. ^55,56^. We show that the reaccumulation phase does not necessarily involve a single collapse for which fragment shapes can play a role in the final outcome, but rather the interactions between precursor aggregates which gravitationally reaccumulate to form the final rubble pile. Although the shape of individual components may still have a role in this case, our modeling accounts for the various kinds of frictional forces between spherical components during precursor aggregate collisional interactions, which dominate in the final phases of a reaccumulation event.

### Improvements in gravitational phase modeling: SSDEM

Another improvement in the modeling of the reaccumulation came from the implementation of the SSDEM in pkdgrav^21^. It was first used to simulate successfully the formation of the bilobate shape of the comet 67P by disruption and reaccumulation^57^. SSDEM allows the explicit modeling of the contact forces between particles, parameterized by various friction parameters adjusted to represent realistic material types. SSDEM is thus the most realistic method to compute the reaccumulation, but it comes with a higher cost in computational time. A technique was recently developed and demonstrated to execute the handoff of the output of the fragmentation phase computed by SPH simulations to the input of the reaccumulation phase computed by pkdgrav^20^. With this technique, which is the one used in this paper, it is possible to reconfigure the output of SPH simulations in order to use the more computationally expensive SSDEM code to model more accurately particle collisions and compute the reaccumulation process more efficiently.

### SSDEM simulation parameters

The coefficients of normal and tangential restitution of particles are fixed at *ε*_n_ = 0.5 and *ε*_t_ = 0.5, respectively. This corresponds to moderately dissipative collisions between particles. Since SSDEM models treat particle collisions as reactions of springs due to particle overlaps, the magnitudes of the normal and tangential restoring forces are determined by the spring constants *k*_N_ and *k*_T_ = 2/7*k*_N_. The values of these parameters are described in ref. ^20^.

In order to evaluate the relative effects of the various interparticle frictions accounted for in SSDEM, we performed simulations for three sets of friction parameters. The fiducial friction parameters are based on the measured minimum angle of repose for Bennu^9^. These are compared to a no-friction case and a high friction case using friction coefficient values obtained by Jiang et al.^58^ for rough sand. We take the coefficients of friction and shape factor used in those studies, but decrease the static friction by half. The set of parameter values for this material type are reported in Supplementary Table 1, where we tabulate the values of static friction, *μ*_S_, rolling friction, *μ*_R,_ twisting friction, *μ*_T_, and a shape factor for rolling friction, *β*.

The total simulated time is set to at least 100 times the dynamical time for the system, for a total simulation time of at least 50 h, beyond which aggregates have completed their reaccumulation.

## Supplementary information

Supplementary Information

Peer Review File

Description of Additional Supplementary Files

Supplementary Movie 1

Supplementary Movie 2

Supplementary Movie 3

Supplementary Movie 4

Supplementary Movie 5

## Data Availability

The data that support the findings of this study are available from the corresponding authors upon reasonable request.

## Code availability

The simulations of the fragmentation phase are performed with a custom SPH code for which the details can be found in refs. ^19,45,47^ (and references therein). The simulations of the reaccumulation phase are performed by the code pkdgrav in its custom version that includes the Soft Sphere Discrete Element Method, for which the details can be found in refs. ^20,56^ (and references therein).

We have provided a compiled version of pkdgrav as well as the necessary files required to reproduce the outcome of Case 1, as an example. The input data for other cases presented in this paper are available on reasonable request.
