# Supplementary material for: Collisional formation of top-shaped asteroids and implications for the origins of Ryugu and Bennu
Source: Nat Commun. 2020 May 27;11:2655. doi: 10.1038/s41467-020-16433-z (PMC7253434; doi:10.1038/s41467-020-16433-z)
Supplement: Supplementary file 1 — Supplementary Information [file 41467_2020_16433_MOESM1_ESM.pdf]

Supplementary Information

Collisional formation of top-shaped asteroids and implications for the origins of Ryugu and Bennu

Michel and Ballouz et al.

Supplementary Figures

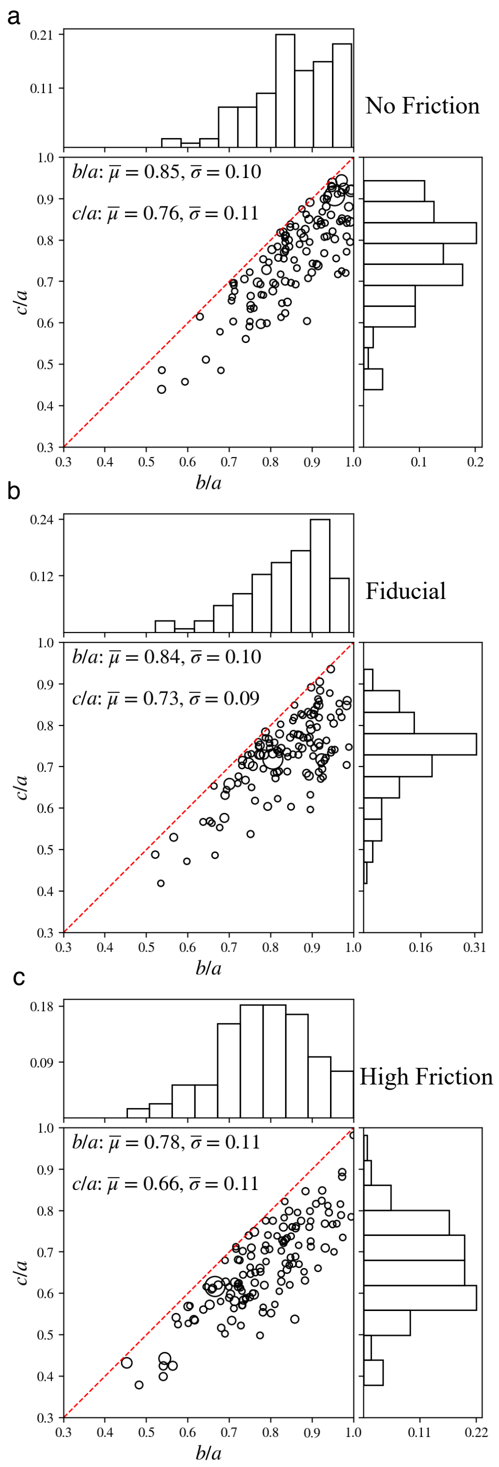

Supplementary Fig. 1 | Inter-particle friction influences aggregate shape. For the case of v_{imp} = 5 km/s , θ_{imp} = 15, R_{imp} = 7 km, we show the axial ratios of each reaccumulated remnant for three different simulations where the inter-particle friction is varied: **a** No friction; **b** Fiducial; **c** High friction (see Supplementary Table 1 for the corresponding parameters). Increasing the effective inter-particle friction results in more elongated prolate shapes, while having no friction results in more spherical shapes.

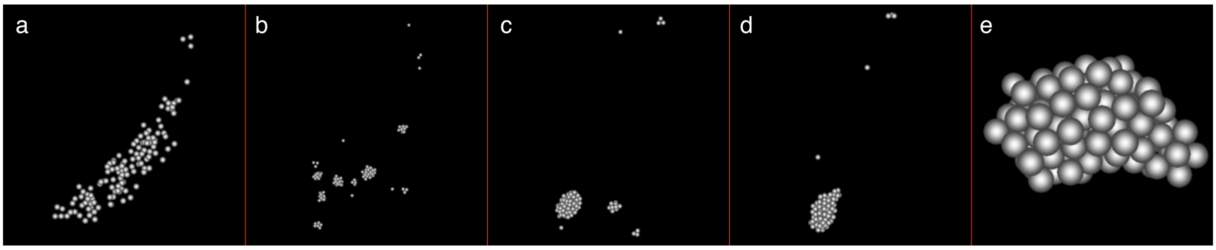

Supplementary Fig. 2 | Slow mergers of aggregates result in elongated objects. Panels a-e show time evolution of the reaccumulation process in simulations centered on a single forming aggregate at time steps of 1 minute, 30 minutes, 2 hours, 3 hours, and 8 hours after the collision. In contrast to the formation of oblate spheroids, the formation of individual equal-sized aggregates that subsequently merge at slow speeds can form prolate elongated objects that more closely resemble the S-type asteroids (433) Eros and (25143) Itokawa.

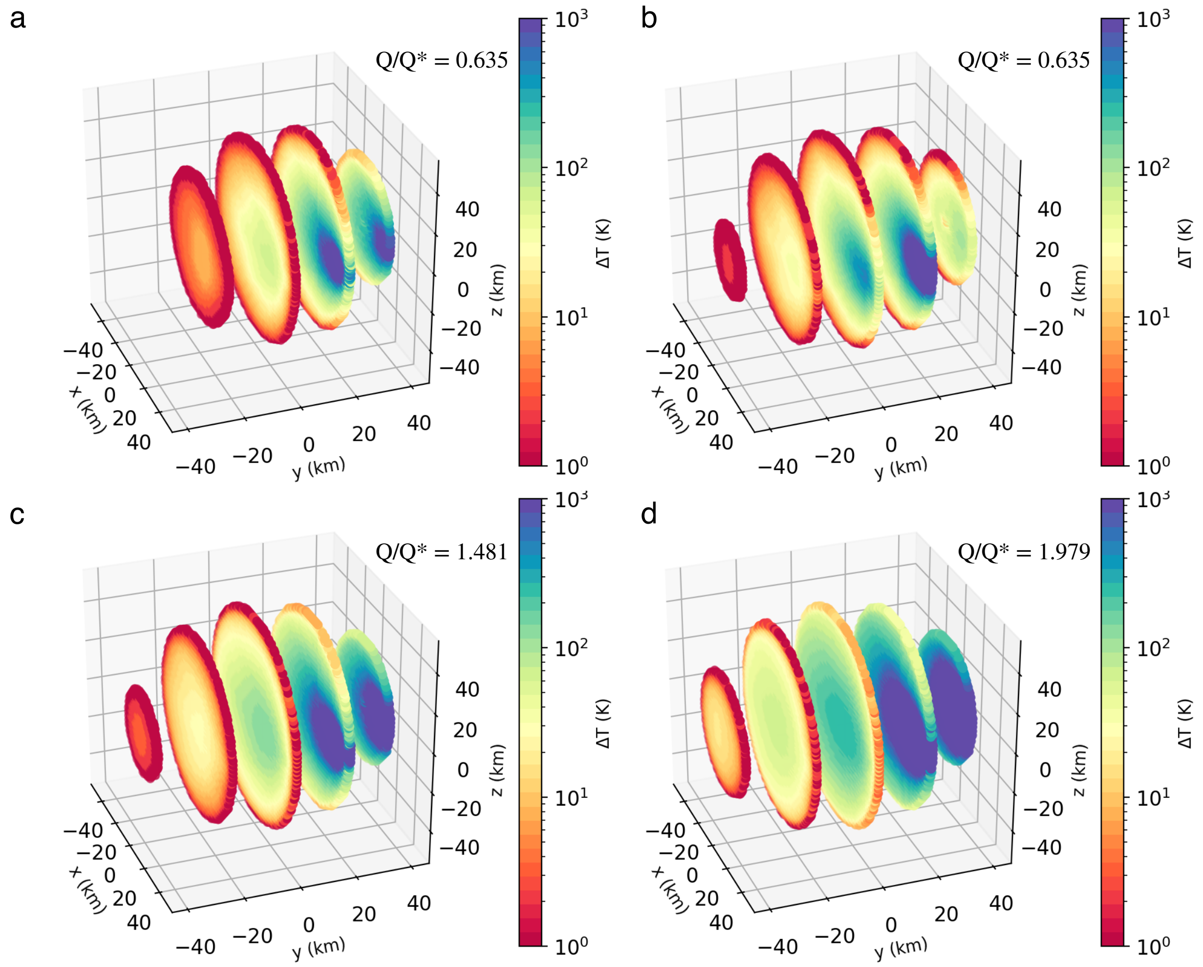

Supplementary Fig. 3 | Visualizations of heating in the parent body, ΔT. This figure shows the change for several slices of the parent body, demonstrating the influence of the impact at heating up the material during catastrophic disruption. From **a** to **d**, the impact energy Q is increased from below the impact energy threshold for disruption Q* (**a**) to above Q* (**b**, **c**, **d**). For each of these four cases, the parent body is at the origin of the reference frame, and the impact direction is along the negative *x* axis.

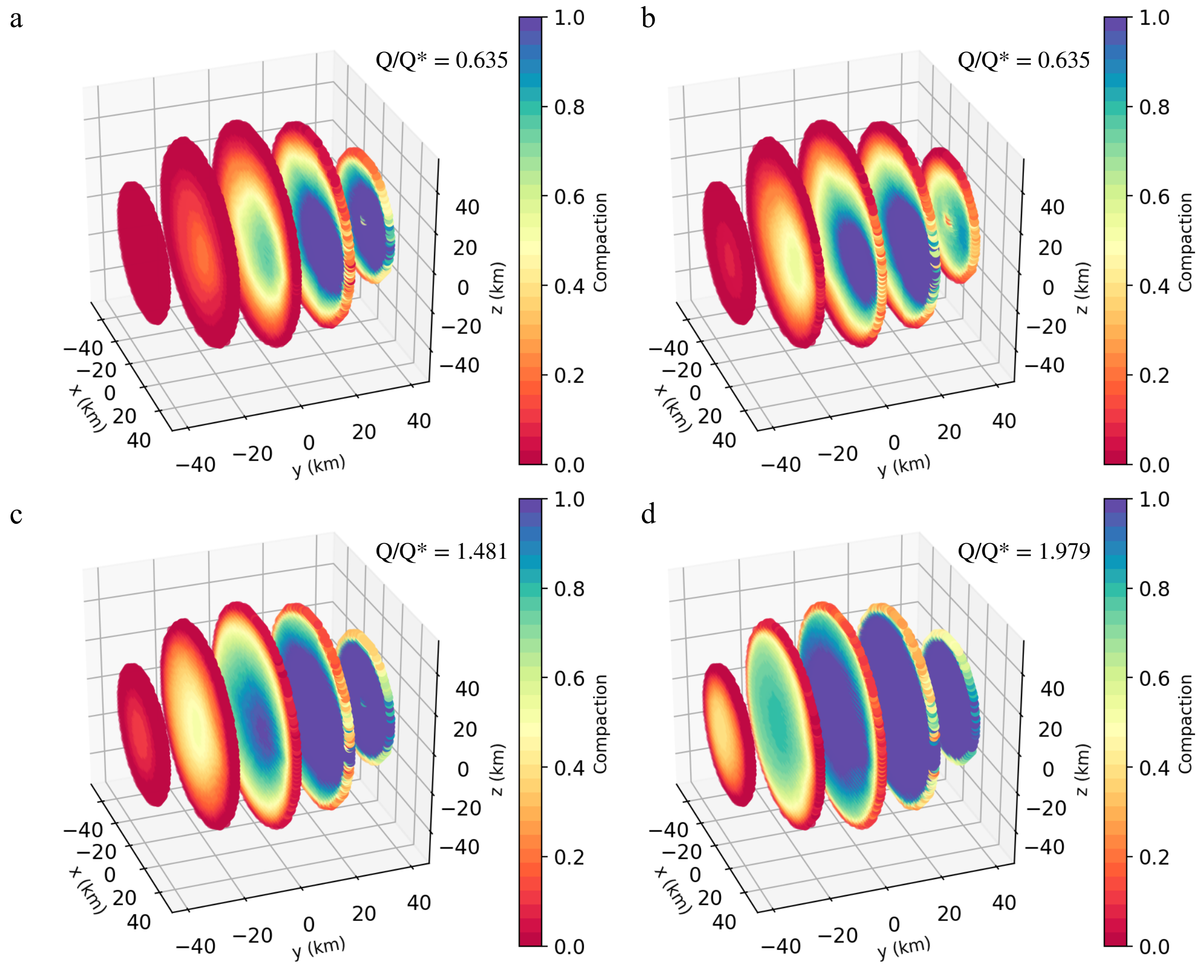

Supplementary Fig. 4 | Visualizations of compaction in the parent body. They demonstrate the influence of the impact at compacting the material during catastrophic disruption. From **a** to **d**, the impact energy Q is increased from below the impact energy threshold for disruption Q* (**a**) to above Q* (**b**, **c**, **d**). For each of these four cases, the parent body is at the origin of the reference frame, and the impact direction is along the negative *x* axis.

Supplementary Table 1. Summary of SSDEM parameters. The fiducial case represents the set of parameters used in the simulations presented in the main text.

Case	ε_n	ε_t	μ_S	μ_R	μ_T	β
Fiducial	0.55	0.55	0.3	0.0	0.0	0.0
No Friction	0.55	0.55	0.0	0.0	0.0	0.0
High Friction	0.55	0.55	0.5	1.31	1.0	0.5
