# Supplementary material for: Collisional formation of top-shaped asteroids and implications for the origins of Ryugu and Bennu
Source: Nat Commun. 2020 May 27;11:2655. doi: 10.1038/s41467-020-16433-z (PMC7253434; doi:10.1038/s41467-020-16433-z)
Supplement: Supplementary file 3 — Description of Additional Supplementary Files [file 41467_2020_16433_MOESM3_ESM.pdf]

Manuscript: **NCOMMS-19-3995858C**

Description of Additional Supplementary Files

File Name: Supplementary Movie 1
Description: Stereoscopic pair movie showing the gravitational phase of the disruption of the parent body for Simulation 3. The movie is centered on the largest remnant following reaccumulation. The color scale and viewing geometry are the same as in Fig. 3a.

File Name: Supplementary Movie 2
Description: Stereoscopic pair movie showing the gravitational phase of the disruption of the parent body for Simulation 3. The movie is centered on the largest remnant following reaccumulation. The color scale and viewing geometry are the same as in Fig. 3b.

File Name: Supplementary Movie 3
Description: Stereoscopic pair movie showing the dynamical Path 1 indicated in Fig. 4 of the manuscript leading to a top-shape aggregate as a result of reaccumulation of particles from the disruption of a parent body.

File Name: Supplementary Movie 4
Description: Stereoscopic pair movie showing the dynamical Path 2 indicated in Fig. 4 of the manuscript leading to an oblate spheroidal aggregate as a result of reaccumulation of particles from the disruption of a parent body.

File name: Supplementary Movie 5
Description: Stereoscopic pair movie showing the dynamical Path 3 indicated in Fig. 4 of the manuscript leading to an oblate spheroidal aggregate as a result of reaccumulation of particles from the disruption of a parent body.
