# Supplementary material for: Collisional formation of top-shaped asteroids and implications for the origins of Ryugu and Bennu
Source: Nat Commun. 2020 May 27;11:2655. doi: 10.1038/s41467-020-16433-z (PMC7253434; doi:10.1038/s41467-020-16433-z)
Supplement: Supplementary file 2 — Peer Review File [file 41467_2020_16433_MOESM2_ESM.pdf]

Reviewers' comments:

Reviewer #1 (Remarks to the Author):

Dear Editor,

I have read with interest the paper “Formation of top-shaped asteroids by collisional disruption: implications for the origin of Ryugu and Bennu” by Michel et al.

In this paper the authors investigate the question of the origin of the top-shape of the Benu and Ryugu asteroids, that are both currently visited by sample-return space mission. Constraining their origin is very important because it will allow to better understand their evolution and how they acquire their today composition, that will be directly measured in laboratory upon come-back to Earth of the different samples, in the next years. As stated in the paper, one of the most puzzling characteristics of these two objects is that they show a proeminent equatorial ridge, that is heavily cratered, indicating that they acquire their ridges very rapidly after their formation. So the question is how is it possible ? and also what is the cause of the strong measured difference in their hydration level ?

In order to solve these mysteries, the author show, using N-body contact-spheres models (the PKD grav code) study the gravitational re accumulation of fragments after the catastrophic disruption of a parent asteroid. They show that a substantial fraction of the remnants may have directly reaccumulated with an oblate shape. That is the main result of the paper. Then the rapid action of the YORP effect, may have given to them their top-shape. Then the author try to estimate the maximum heating temperature during the reaccumulation process. They show that depending on the relative original location of the reaccumulated material with respect to of the impact point, a wide range of peak temperature may be achieved, and thus, which may explain the variety of dehydration level observed. They also discuss compaction effect. They conclude that Benu and Rygu may both have formed in a catastrophic disruption event, enethough they may have experienced different thermal histories.

I think this is a very interesting and timely paper in the current context of space exploration, because it gives a new perspective to understand the origin of these top-shaped objects, and it was a major surprise, and it was not expected, that both Ryugu and Benu have the same shape. This paper explains how a same, and very common mechanism, may naturally result in the observed shape. In addition, it tries to do an interesting link between evolutionary processes (disruption) and surface composition.

I have only minor comments on the paper, mainly clarifications in particular concerning compaction and temperature calculation.

Best Regards,

Sébastien CHARNOZ

****

Specific comments

Title vs. abstract : in the title you talk about “top shape”, but in the abstract you talk about “oblate” object. Please clarify the link between the two in the abstract.

Lines 82 to 91 : What is an “individual component” ? a particle ? clarify

In this paragraph we would like to have more info about the particle size, how it compares to the size of the final reaccumulates (or how many particle they contain). What motivates the choice of the 18° friction angle. A discussion on the effect of this repose angle would be good, as it is not clear how the results depend on this choice.

Lines 99 to 102: “It should not tae much time” : this is pretty vague. Can you clarify what enters in the calculation of the YORP effect timescales? What makes this timescale so short ?

Line 102 : Authors are talking about “oblate” shape, then “top-shape”. What makes the distinction? It gives the feeling that both concepts are the same, but clear distinction is made elsewhere in the paper. I think that the authors should clearly define what “oblate “ and “top shape” mean in the paper, even though, I guess the frontier between the two may be fuzzy at some point

Figure 2 : Very difficult to read. Labels and numbers are almost unreadable.

Line 165 : Oblate is defined as c/a>0.75. But if c/a=1 it is spherical? and if c/a>1 it is prolate? Can you clarify? And in particular I think this is important when F is computed: spherical and prolate objects should not be considered when computing the fraction of oblate objects.

Compaction C ?: I have to admit that I did not understood at all how compaction C is calculated. The paper says : “degree of compaction at the center (Ccenter), where the center is defined as the fraction of particles within a radius of 5 km from the center of mass of the parent body”, but I do not understand it.

Legend of Figure 3 : It is hard to link the text, explaining the reaccumualtion process, to the images. In particular, if a disk is seen, could you clear show the disk in the image witn an arrow (for example
?) or colored particle s?

Temperature calculation : I do not understand how temperature change is calculated, in PKDgrav, that has no equation of state. I guess maybe this calculation comes from the impact simulation, but if I remember well, Tillotson EOS does not really gives the temperature. Can you clarify this point?

In particular when computing temperature, I guess this is equilibrium temperature, however during the shock, the thermodynamics is not at equilibrium. So what is the meaning of the computed temperature? Can you discuss that?

END

Reviewer #2 (Remarks to the Author):

The submitted manuscript investigates the origin of 'top-shape' near Earth asteroids coupled to difference in hydration levels as found in recently visited NEAs Ryugu (JAXA) and Bennu (NASA). This work is very ambitious as it tries to solve two non-trivial problems of current asteroid science with one single process: post-impact re-accumulation of a main belt parent body. Moreover, the possibility that two such bodies come from one single disruption is introduced. This study deserves attention indeed and may pioneer future investigation on this topic.

Nevertheless, this reviewer finds that many parts of the manuscript are somewhat speculative, and concerns about the method and conclusions not supported by enough compelling arguments, as detailed below. The scenario described by the authors is one of the possible explanations to this problem, but they do not provide convincing evidence to prefer their solution over alternatives.

Technically sound data and strong evidence for conclusions are main criteria for publication in Nature Communications. Unfortunately, trouble with part of utilized methods and no strong evidence for conclusions prevents this reviewer from recommending the publication of this research in Nature Communications.

However, putting forward challenging ideas and theories is a must in science progress and it is good to have a chance to make other researchers pay attention to new visions. For this reason, I am sure that at least part of the submitted research may find suitable publication in other specialized journals.

Instead, this reviewer finds that the study concerning material heating during shattering phase is very interesting. Were the ansatz dropped of having twin generation of the two rubble-piles, the study looks indeed very promising to help and understand different levels of hydration in observed NEAs. I encourage the authors to follow that innovative path which is potentially capable to shed light to interpretation of new observations.

Major worries about the submitted manuscript are pointed out here.

1) Concerns on twin origin of Ryugu and Bennu.

A number of lucky circumstances need to combine:

a) Two bodies, with similar shape and different size, are produced in the main belt after the catastrophic collision of a single common parent body, based on SPH+N-body numerical simulations.

b) Both bodies end up as NEAs after reaching suitable MMR through Yarkovsky drift and surviving potential collisional disruption in the main belt.

c) Both bodies happen to have orbits and Delta(V) such that two space agencies target them for selected space missions.

Did the authors try to estimate the probability of such a chain of events to occur? (a) and (b) could be softened in the case that tens or hundreds of similar rubble-piles are created in the proposed single collision, however the authors do not mention how many similar bodies are typically created in their numerical simulations. Though (c) may still be a challenge to work around.

- The authors state (‘Results’):

“In addition, Bennu and Ryugu have been linked to asteroid families in the inner main belt that have dynamical ages of 1 Ga [12]. If the ridges on those bodies formed at approximately the same time,
they would be subject to a billion years of impacts from main belt projectiles.”

On one hand, NEAs dynamical time in the main belt before they reach strong resonaces through Yarkovsky drift is of the order of tens to ~100 Ma, as shown by CRE analysis in late ’90. Only iron meteorites show CRE ages close to 1 Ga. On the other hand, were Ryugu and Bennu created 1 Ga ago, they would both hardly survive on their way to a resonance, given that collisional lifetimes in the main belt are less than 500 and 350 Ma, respectively for their sizes (estimating collision lifetimes from Farinella and Vokrouhlicky, 1999).

- On the utilized methods:

The authors state:

“We performed a series of simulations of disruptions of 100-km-diameter asteroids with microporosity, as expected for parent bodies of dark (geometric albedo < 0.1) asteroid families [17],…”

Reference [17] suggests microporosity might be present in parent bodies of CC meteorites and they measure it for such meteorites to be up to some 20%. However, Reference [19] used material properties of porous targets that provided the best match to impact experiments on pumice targets, that typically have porosity larger than 65% (70% in the material chosen by [19]). Crush-up curves of CC meteorites are not necessarily represented by that of pumice, thus flawing the method used.
Current SPH simulations may be good to represent damage on very porous pumice-like target. However, pumice does not presumably match any of the known asteroid materials: even CC meteorites have porosities way lower than pumice.

To date, no comparison of high-speed laboratory shattering experiments and SPH results is known by this referee to have been published for materials other than pumice (Ref. [19]). Typically, SPH simulations of asteroid shattering on non-porous targets show extremely high damage (sometimes down to resolution size) with the formation of a somewhat small largest fragment and a swarm of equal small size fragments. That pattern does not match what laboratory impact experiments show for low micro-porosity materials. Therefore, it is questionable to what extent SPH results can be currently trusted for shattering of non-high-porosity asteroids.

2) Concerns on top-shape formation.

Some of the statements in the ‘Introduction’ section look speculative, not supported by strong evidence and instead seem to drive the whole paper.

“However, the equatorial ridges of both Ryugu and Bennu appear to be their oldest surface features, predating the formation of large equatorial craters [5,6,1]. These geologic characteristics suggest that Ryugu and Bennu formed directly as top-shapes, or achieved such a shape early after their
formation.”

Both Ryugu and Bennu show their largest crater-like structures right on their equatorial ridge. It is at least statistically suspicious that both bodies underwent their largest impacts --- after formation--- in the 'equatorial' region. Other NEAs (e.g. 2008 EV5) show similar features according to shape models derived from radar observation. It cannot be ruled out that such features correspond to locations of large boulders landed at the end of the re-accumulation process (this is at least at the same speculative level as authors' ansatz). Once in the inner planet region, the YORP effect may have spun up such bodies to the point that the ridge formed and boulders on equatorial region were lost or lofted and re-landed off-equator, due to centrifugal force being larger than gravitational attraction at that location. In fact, no large boulders are currently located on the ridge of these bodies, but many are scattered at larger latitudes. Current spin rate for Ryugu is way below threshold for
instability. Bennu's spin period (4.276 hr) is instead not that far from typical periods for instability corresponding to low density (C/B-type) asteroids. Both rotation states may have been reached from higher spin rates after tidal interaction with some former satellite.

The occurrence of oblate shapes in formed rubble-piles does not imply necessarily the formation of a ridge on its own. No compelling evidence for the formation of a ridge while in the main belt is offered by simulations, instead this is left to YORP evolution based on former studies on ridge (and satellite) formation for NEAs. The argument seems weak.

3) Other concerns.

The authors state that:

"These observations suggest that the two bodies have different levels of hydration, assuming that the mineralogy of their surfaces is representative of their bulk compositions."

This looks like a strong assumption indeed, not supported by any evidence. Different mineralogical features on the surface may well correspond to impacts of bodies with different composition and hydration levels. A few, or even just one sub-catastrophic collision by a strongly hydrated body may provide enough material to garden the target suitably.

- Missing data in 'Results'?

According to sentence:

"… using the Soft-Sphere Discrete Element Method (SSDEM) and assuming specific sets of values of friction parameters to compute the contact forces between the particles reaccumulating to form aggregates (Methods)."

Values for such friction parameters should be provided. The available version of the manuscript does not include any value of friction parameters in Methods to be checked.

<u>Answers to Reviewers</u>

We thank the reviewers for their insightful comments and corrections.

Below, we address their concerns point by point, and highlight implemented changes in the manuscript, if they occur.

<u>Reviewer 1.</u>

Title vs. abstract: in the title you talk about "top shape", but in the abstract you talk about "oblate" object. Please clarify the link between the two in the abstract.

Thank you for pointing this out. We attempt to clarify the relationship between "oblate objects" and "top-shapes" by merging two sentences in the abstract into:

"Here we show, through numerical simulations of large asteroid disruptions, that oblate spheroids, some of which have a pronounced equator defining a spinning top shape, can form directly through gravitational reaccumulation."

Lines 82 to 91 : What is an "individual component" ? a particle ? clarify

By components we mean particles in the simulations.

We have changed component to particle to clarify.

In this paragraph we would like to have more info about the particle size, how it compares to the size of the final reaccumulates (or how many particles they contain). What motivates the choice of the 18° friction angle. A discussion on the effect of this repose angle would be good, as it is not clear how the results depend on this choice.

18° was chosen as it conforms with the angle of repose of Bennu's surface from detailed measuring of its shape properties in Barnouin et al [2019]. This is stated at the end of the paragraph:
"friction coefficients commensurate with a global aggregate angle of friction of 18° (see the Methods for specific parameters and discussion), which is consistent with the angle of friction estimated for Bennu [Barnouin et al. 2019]."

We point to Supplementary Fig. 1, which shows the effect of changing the angle of friction. The caption reads:

“Inter-particle friction influences the final shapes formed by the gravitational reaccumulation process**.** For the case of v_{imp} = 5 km/s , θ_{imp} = 15, and R_{imp} = 7 km, we show the axial ratios of each reaccumulated remnant for three different simulations where the inter-particle friction is varied (see Supplementary Table 1). Increasing the effective inter-particle friction results in more elongated prolate shapes, while having no friction results in more spherical shapes.”

We omitted pointing to this figure in the main text. We now do so, by stating:

“Increasing the angle of friction tends to lead to a larger fraction of aggregates having more prolate shapes (Supplementary Fig. 1).”

Lines 99 to 102: “It should not take much time”: this is pretty vague. Can you clarify what enters in the calculation of the YORP effect timescales? What makes this timescale so short?

The calculation of the YORP timescale is explained in the references. The calculation goes beyond the scope of this paper. The timescale is proportional to the size of object. Larger objects, such as Vesta and Ceres, are not affected.

Furthermore, the current YORP timescale on Bennu has been measured directly to be ~ 1.5 Myr [Hergenrother et al. 2019]. For a similar shape, it’s YORP timescale in the main belt would be ~ 4 Myr.

“After the formation of an oblate spheroid, it should not take much time (order of 10^4 to10^6 years for kilometer-sized asteroids [3,23,24]) for post-processes such as YORP to lead to top shapes such as those currently observed. Measurements of Bennu’s YORP acceleration show that its spin-rate doubling period is roughly 1.5 Myr at its current orbit [25]. In the inner main belt, this timescale would be ~ 4 Myr.”

Line 102: Authors are talking about “oblate” shape, then “top-shape”. What makes the distinction? It gives the feeling that both concepts are the same, but clear distinction is made elsewhere in the paper. I think that the authors should clearly define what “oblate“ and “top shape” mean in the paper, even though, I guess the frontier between the two may be fuzzy at some point.

As we state in the abstract, a top-shape asteroid is an oblate object with a pronounced equatorial ridge.

Figure 2: Very difficult to read. Labels and numbers are almost unreadable.
Line 165: Oblate is defined as c/a>0.75. But if c/a=1 it is spherical? and if c/a>1 it is prolate? Can you clarify? And in particular I think this is important when F is computed: spherical and prolate objects should not be considered when computing the fraction of oblate objects.

We have updated Fig. 2 to become easier to read:

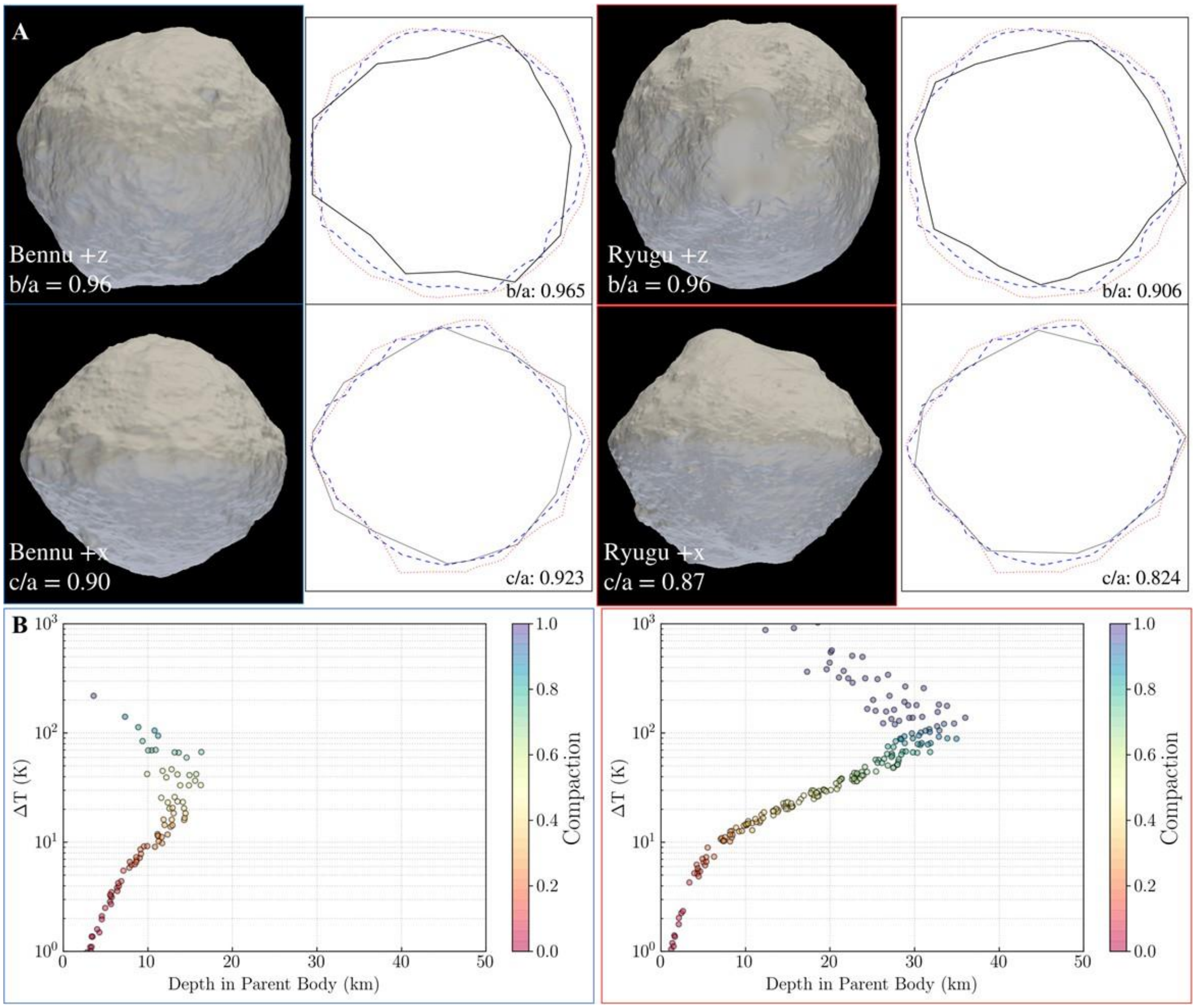

The value of c/a ranges from 0 to 1.
For c/a < 0.5, an object is prolate. As c/a approaches 1, the object is more spherical.

In order to determine the relative amount of oblate reaccumulated aggregates in our simulations, we set a limit to oblateness as c/a > 0.75.

We are confused by the comment: “And in particular I think this is important when F is computed: spherical and prolate objects should not be considered when computing the fraction of oblate objects.”

F represents the fraction of oblate objects (c/a>0.75). If spherical and prolate objects are not considered in the computation, then F=1 for all cases, which would make it a useless metric.

Compaction C ?: I have to admit that I did not understand at all how compaction C is calculated. The paper says: “degree of compaction at the center (C_{center}), where the center is defined as the fraction of particles within a radius of 5 km from the center of mass of the

parent body", but I do not understand it.

As we state in the section: "*Impact-driven hydration diversity in rubble piles"*

"Compaction measures the increase in the density of the individual particle relative to its initial uncompacted state."

And in the caption of Table 1, we attempt to elaborate further by stating:

"An object is fully compacted (no microporosity) when $C = 1$ and has its original microporosity when $C = 0$."

In Table 1, we show the value of ΔT_{center} and C_{Center}, which we define as the mean change in temperature and compaction of the set of particles that are originally within 5 km of the center of the parent body, respectively.
We define these metrics, in order to provide a quick-look comparison of the degree of alteration induced by the family-forming impact far enough away from the impact point, in a region that is expected to have the highest degree of alteration from internal parent body processes.

We attempt to clarify any ambiguity by slightly revising the caption in Fig. 1 to now say:

"An object is fully compacted (no microporosity) when $C = 1$ and has its original microporosity when $C = 0$. The center is defined as the subset of material originally within a radius of 5 km from the center of mass of the parent body."

Legend of Figure 3: It is hard to link the text, explaining the reaccumualtion process, to the images. In particular, if a disk is seen, could you clearly show the disk in the image with an arrow (for example?) or colored particles?

We thank the reviewer for the comment. We have changed Fig. 3c to now include arrows that highlight features of interest. The caption for Fig. 3c now reads

"Example snapshots of three cases of the gravitational reaccumulation of those smaller aggregates. Path 1 shows the ejection of a material stream that forms an elongated disk (blue arrows) with a dense core (red arrow). The disk of material then accretes onto the equator, spinning up the core through conservation of angular momentum and building up a ridge (green arrow). Paths 2 and 3 show the formation of oblate spheroids through the collapse of multiple cores that subsequently coalesce. In the case of path 2, the aggregates have a high enough relative speed that the smaller bodies break up on impact and individual particles accrete onto the larger body (blue arrow) isotropically. In the case of path 3, a kind of nucleation occurs, where a large primary aggregate quickly forms out of the merger and becomes the focus point of a gradual deposition of smaller aggregates that accrete isotropically, slowly building up an oblate spheroid (see Supplementary Movies 1, 2, and 3 of these three paths) [40]."

Temperature calculation: I do not understand how temperature change is calculated, in pkdgrav, that has no equation of state. I guess maybe this calculation comes from the impact simulation, but if I remember well, Tillotson EOS does not really gives the temperature. Can you clarify this point?

Effectively, the temperature change is calculated in the first step by the SPH simulations, the output of which is then passed on to pkdgrav to simulate the gravitational reaccumulation phase.

The Tillotson EOS is used in the SPH simulation, which does not directly compute the temperature and rather the internal energy. However, there is a relationship between temperature and internal energy through an assumption on the heat capacity, which has already been used in past studies [Schwartz et al. 2018].

One improvement in our current estimate is that we account for the dependence of the heat capacity on temperature, to convert internal energy to temperature [Jutzi & Michel 2019, in Review]. We discuss these details in the Methods section, under "Improvements in modeling material impact heating and compaction", where we state:

"The shock physics code calculations of the collisions were performed in [Jutzi et al. 2019] using the relatively simple Tillotson equation of state, which does not allow for a direct computation of a thermodynamically consistent temperature. However, we can still approximately compute the temperature. Such a computation was improved [Jutzi & Michel 2019, in Review] by analyzing the increase in specific internal energy to model the change in temperature caused by an impact. As shown in [Emsenhuber et al. 2018], this approach leads to temperatures comparable to the ones computed via the more sophisticated ANEOS equation of state, as long as vaporization is negligible."

In particular when computing temperature, I guess this is equilibrium temperature, however during the shock, the thermodynamics is not at equilibrium. So what is the meaning of the computed temperature? Can you discuss that?

The reviewer makes a good point; however, as stated above we get comparable temperature changes to simulations with more complex equations of state. This represents the state-of-the-art in numerical modeling of instantaneous impact-induced temperature changes. Future work that will address the potential kinematics of post-impact cooling will require a more detailed treatment that is out of the scope of this current study.

END

Reviewer #2 (Remarks to the Author):

The submitted manuscript investigates the origin of 'top-shape' near Earth asteroids coupled to difference in hydration levels as found in recently visited NEAs Ryugu (JAXA) and Bennu (NASA). This work is very ambitious as it tries to solve two non-trivial problems of current asteroid science with one single process: post-impact re-accumulation of a main belt parent body. Moreover, the possibility that two such bodies come from one single disruption is introduced. This study deserves attention indeed and may pioneer future investigation on this topic.

Nevertheless, this reviewer finds that many parts of the manuscript are somewhat speculative, and concerns about the method and conclusions not supported by enough compelling arguments, as detailed below. The scenario described by the authors is one of the possible explanations to this problem, but they do not provide convincing evidence to prefer their solution over alternatives.
Technically sound data and strong evidence for conclusions are main criteria for publication in Nature Communications.

Unfortunately, trouble with part of utilized methods and no strong evidence for conclusions prevents this reviewer from recommending the publication of this research in Nature Communications.

However, putting forward challenging ideas and theories is a must in science progress and it is good to have a chance to make other researchers pay attention to new visions. For this reason, I am sure that at least part of the submitted research may find suitable publication in other specialized journals.

Instead, this reviewer finds that the study concerning material heating during shattering phase is very interesting. Were the ansatz dropped of having twin generation of the two rubble-piles, the study looks indeed very promising to help and understand different levels of hydration in observed NEAs. I encourage the authors to follow that innovative path which is potentially capable to shed light to interpretation of new observations.

Major worries about the submitted manuscript are pointed out here.

1) Concerns on twin origin of Ryugu and Bennu.

A number of lucky circumstances need to combine:

a) Two bodies, with similar shape and different size, are produced in the main belt after the catastrophic collision of a single common parent body, based on SPH+N-body numerical simulations.
b) Both bodies end up as NEAs after reaching suitable MMR through Yarkovsky drift and surviving potential collisional disruption in the main belt.
c) Both bodies happen to have orbits and Delta(V) such that two space agencies target them

for selected space missions.

Did the authors try to estimate the probability of such a chain of events to occur? (a) and (b) could be softened in the case that tens or hundreds of similar rubble-piles are created in the proposed single collision, however the authors do not mention how many similar bodies are typically created in their numerical simulations. Though (c) may still be a challenge to work around.

Firstly, we thank the reviewer for his close reading and insightful comments on our manuscript. We would challenge the conclusion that a twin-generation is unlikely, based on the following well-established lines of evidence for the generation of small km-size asteroids in the main belt, and their migration to the inner Solar System via dynamical paths.

The number of small km-size bodies that form from the catastrophic disruption of a 100-km size parent body is quite large, on the order of ~ 10^4-10^5 individual asteroids (e.g., Jutzi et al. 2019). After their formation, roughly half end up drifting inwards to a resonance that would inject them into Near-Earth space.

Just because these bodies are accessible doesn’t mean that there are other NEAs from the same family that are not reachable by space missions. Once you enter a resonance and start crossing the orbits of the Earth, the trajectories are chaotic and highly dependent on encounters with the inner planets, such that they dynamically decouple from their source regions.

For the same family you may have objects with totally different orbits from that of Bennu and Ryugu. If we start with 10^5 asteroids, for which a significant fraction is accessible from Earth, having two is not so implausible.

In comparison, most NEA S-types likely come from the same family, Flora, and these objects have very different orbits, but there are many individual cases of multiple objects having accessible orbits.

- The authors state (‘Results’):

“In addition, Bennu and Ryugu have been linked to asteroid families in the inner main belt that have dynamical ages of 1 Gya [12]. If the ridges on those bodies formed at approximately the same time, they would be subject to a 1 billion years of impacts from main belt projectiles.”

On one hand, NEAs dynamical time in the main belt before they reach strong resonaces through Yarkovsky drift is of the order of tens to ~100 Ma, as shown by CRE analysis in late ’90. Only iron meteorites show CRE ages close to 1 Ga. On the other hand, were Ryugu and Bennu created 1 Ga ago, they would both hardly survive on their way to a resonance, given that collisional lifetimes in the main belt are less than 500 and 350 Ma, respectively for their sizes (estimating collision lifetimes from Farinella and Vokrouhlicky, 1999).

While the derived collisional lifetimes for km-size objects are indeed 350-500 Myr, these represents the characteristic timescale associated with the decay rate of all objects created from the family forming collision (e.g., Bottke et al. 2005). Therefore, it is not expected that all objects would be disrupted in this time-frame. Indeed, one would expect ~ half of the 10^5 objects originally created by the collisional disruption of the parent to have survived. It is plausible then, that Bennu and Ryugu represent survivors of the main belt collisional disruption of this population as they migrated inward.

- On the utilized methods:

The authors state:

“We performed a series of simulations of disruptions of 100-km-diameter asteroids with microporosity, as expected for parent bodies of dark (geometric albedo < 0.1) asteroid families [17],…”

Reference [17] suggests microporosity might be present in parent bodies of CC meteorites and they measure it for such meteorites to be up to some 20%. However, Reference [19] used material properties of porous targets that provided the best match to impact experiments on pumice targets, that typically have porosity larger than 65% (70% in the material chosen by [19]). Crush-up curves of CC meteorites are not necessarily represented by that of pumice, thus flawing the method used. Current SPH simulations may be good to represent damage on very porous pumice-like target. However, pumice does not presumably match any of the known asteroid materials: even CC meteorites have porosities way lower than pumice.

To date, no comparison of high-speed laboratory shattering experiments and SPH results is known by this referee to have been published for materials other than pumice (Ref. [19]). Typically, SPH simulations of asteroid shattering on non-porous targets show extremely high damage (sometimes down to resolution size) with the formation of a somewhat small largest fragment and a swarm of equal small size fragments. That pattern does not match what laboratory impact experiments show for low micro-porosity materials. Therefore, it is questionable to what extent SPH results can be currently trusted for shattering of non-high-porosity asteroids.

We thank the reviewer for the comment, but we will point out that there are indeed other publications that compared SPH simulations to laboratory experiments.

In particular, the SPH code that we employ is an updated version of Benz and Asphaug (1994), which reproduced successfully impacts into basalt targets. These SPH simulations do not necessarily shatter to resolution size, and they are able to accurately reproduce the formation of a core fragment made of a large number of undamaged individual particles and spall fragments, as observed in the experiments.

Even though a simulation of a catastrophic disruption at the asteroid-scale shows a fully damaged target, this does not preclude its accuracy, since we show that, at smaller scales, we are

able to reproduce accurately the level of damage and fragmentation for a variety of different materials.

Furthermore, the crush-curve of the C-type parent body is likely different than individual CC meteorites. Indeed, one has to differentiate between the microporosity found in individual meteorites and the macroporosity (void space) of an entire parent body. For example, based on the measurements of the masses of Bennu and Ryugu the total porosity (micro+macro) can be up to 50-60% [Barnouin et al. 2019, Watanabe et al. 2019].

In fact, the porosity of the parent body used in this simulation is 50%, and not the actual porosity of pumice which, as the reviewer points out, is much higher.

We clarify this point by including more details in the Methods section of the manuscript. It is true that the crush-curve used may not accurately represent CC-meteorite parent bodies. However, it is one (and maybe the only one) that has both been measured and used in impact simulations for validation of those simulations by confrontation with experiments using the same target properties [Jutzi et al. 2009, Icarus 201, 802-813]. Since CC meteorites are relatively rare, their use in destructive experimentation is disallowed. Therefore, porous analogs, such as pumice, are commonly used in the literature to gain a better understanding of the impact response of CC objects in the Solar System [Nakamura 2017, Planet. Space Sci 149].

2) Concerns on top-shape formation.

Some of the statements in the 'Introduction' section look speculative, not supported by strong evidence and instead seem to drive the whole paper.

"However, the equatorial ridges of both Ryugu and Bennu appear to be their oldest surface features, predating the formation of large equatorial craters [5,6,1]. These geologic characteristics suggest that Ryugu and Bennu formed directly as top-shapes, or achieved such a shape early after their formation."

Both Ryugu and Bennu show their largest crater-like structures right on their equatorial ridge. It is at least statistically suspicious that both bodies underwent their largest impacts ---after formation--- in the 'equatorial' region. Other NEAs (e.g. 2008 EV5) show similar features according to shape models derived from radar observation. It cannot be ruled out that such features correspond to locations of large boulders landed at the end of the re-accumulation process (this is at least at the same speculative level as authors' ansatz). Once in the inner planet region, the YORP effect may have spun up such bodies to the point that the ridge formed and boulders on equatorial region were lost or lofted and re-landed off-equator, due to centrifugal force being larger than gravitational attraction at that location. In fact, no large boulders are currently located on the ridge of these bodies, but many are scattered at larger latitudes. Current spin rate for Ryugu is way below threshold for instability.

We agree with the reviewer that a YORP-driven instability of the equator may lead to the lofting of equatorial boulders. However, there is no geologic evidence to support this statement.

During the gravitational reaccumulation process of a rubble-pile, material must reaccumulate at the mutual escape speed of the two bodies – in this scenario, a ~ 1-km size rubble-pile and a 100-m size boulder. This escape speed would amount to a few cm/s. Such an impact would not produce a bowl-shaped crater, indeed it may not even form a visible indentation. Bowl-shaped craters, which are clearly seen on the surface of both asteroids, are created by a hyper-velocity impact. These equatorial craters have raised rims and depth-diameter ratios of ~ 0.15 [Walsh et al. 2019, Barnouin et al. 2019]. Furthermore, there are large craters formed at higher latitudes, with similar morphologies. It would seem overly-complex to require a different mechanism for their formation. As previously stated, the Walsh et al. (2019) paper on the geology of Bennu's surface describes in detail the morphology of these craters on the equator and determined them to have been created from impacts in the main belt.

While subsequent surface evolution in near-Earth space may have led to boulder lofting, and indeed we do see high-latitude bias for large boulder on Bennu, these boulders are perched on the surface of the asteroid and did not form crater-like features.

Therefore, as already concluded by Walsh et al. [2019] and Sugita et al. [2019], the equatorial ridge is indeed the oldest surface feature, as the largest craters overlay it on both asteroids.

Bennu's spin period (4.276 hr) is instead not that far from typical periods for instability corresponding to low density (C/B-type) asteroids. Both rotation states may have been reached from higher spin rates after tidal interaction with some former satellite.
The occurrence of oblate shapes in formed rubble-piles does not imply necessarily the formation of a ridge on its own. No compelling evidence for the formation of a ridge while in the main belt is offered by simulations, instead this is left to YORP evolution based on former studies on ridge (and satellite) formation for NEAs. The argument seems weak.

YORP is not necessarily needed as we do show that gravitational reaccumulation can sometimes from rubble-piles with a pronounced equator in Dynamical Path 1 (Fig. 3c).

However, we do concede that the subsequent action of YORP on an oblate spheroid may be the primary mechanism for the formation of a pronounced equatorial ridge as the fast-spin may lead to down-slope motion to the equator (the equipotential low at high spin-rates). As we state in the manuscript, our simulations are still too coarse to resolve the topographic details at the same level as 1m-scale shape models derived from spacecraft observations of both asteroids.

Nevertheless, as we stress in the manuscript, an oblate or spherical shape is a pre-requisite for formation of a top-shape through YORP spin-up. Indeed, most of the former simulations that show the formation of an equatorial ridge through YORP-action have an oblate sphere as an initial condition.

Dynamical studies of the spins of main belt asteroids show that YORP evolution is required to explain their distribution [Marzazi et al. 2011, Icarus 214]. Moreover, the expected YORP

timescale in the main belt for asteroids that are 1-km in size is expected to be ~ 4 Myr. This provides ample time for the transformation of an oblate spheroid to a top-shape as the formation of the largest craters on Bennu (~100 m diameter) require ~ 50 Myr in the main belt, based on assumed impact rate in the main belt, $P_i \sim 3 \times 10^{-18}$ km^{-2} yr^{--1} [Bottke et al. 2005], and the cratering efficiency on 0.18 MPa strength soil target [Holsapple & Housen 1993] at 5 km/s. In this scenario, subsequent large-scale YORP deformation would be plausible.

Nevertheless, the direct geologic evidence from observations necessitate an alternative mechanism. The geologic evidence shows that large-scale YORP deformation that leads to the formation of equatorial ridge throughs has not happened since the formation of the largest equatorial craters, which post-date the equatorial ridge on both objects.

The novelty of our simulations lies in the fact that shape distributions of the smallest rubble-pile remnants following catastrophic disruption have not been explored in detail before. Our findings show that a large fraction of reaccumulated remnants provide the correct shape or initial conditions to form top-shapes, providing a necessary alternative.

3) Other concerns.

The authors state that:

“These observations suggest that the two bodies have different levels of hydration, assuming that the mineralogy of their surfaces is representative of their bulk compositions.”

This looks like a strong assumption indeed, not supported by any evidence. Different mineralogical features on the surface may well correspond to impacts of bodies with different composition and hydration levels. A few, or even just one sub-catastrophic collision by a strongly hydrated body may provide enough material to garden the target suitably.

Firstly, the hydration level on both asteroids was found to be homogeneous, with no evidence of variable states across different regions [Hamilton et al. 2019, Kitazato et al. 2019].

Therefore, it is impossible that the entire surface is only representative of impact contamination, and the implications of such a mechanism would mean that our understanding of asteroid mineralogy is solely based on the kind of impactor that last hit a surface at a sub-catastrophic level.

Moreover, even in non-disruptive collisions (cratering impacts) the retention rate of projectiles impacting at high speeds (~ 5 km/s for the main belt) is < 10% [Daly et al. 2018]. Therefore, big projectiles would be needed to achieve what the reviewer suggest to be contamination, which would have totally disrupted Bennu.

Furthermore, spectral properties are homogeneous inside and outside of observed craters. This suggests that even if a small portion of a projectile can survive, it does not leave a strong signature in the observed spectra, unless it was the exact composition as the target.

- Missing data in ‘Results’?

According to sentence:

"… using the Soft-Sphere Discrete Element Method (SSDEM) and assuming specific sets of values of friction parameters to compute the contact forces between the particles reaccumulating to form aggregates (Methods)."

Values for such friction parameters should be provided. The available version of the manuscript does not include any value of friction parameters in Methods to be checked.

The values of the friction parameters are already provided. As stated in the Methods section under the sub-title “SSDEM simulation parameters”:

“The set of parameter values for this material type are reported in Supplementary Table 1, where we tabulate the values of static friction, μ_S, rolling friction, μ_R, twisting friction, μ_T, and a shape factor for rolling friction, β.”

Supplementary Table 1 shows:

Supplementary Table 1. Summary of SSDEM parameters. The fiducial case represents the set of parameters used in the simulations presented in the main text.

Case	ε_n	ε_t	μ_S	μ_R	μ_T	β
Fiducial	0.55	0.55	0.3	0.0	0.0	0.0
No Friction	0.55	0.55	0.0	0.0	0.0	0.0
High Friction	0.55	0.55	0.5	1.31	1.0	0.5

Reviewers' comments:

Reviewer #1 (Remarks to the Author):

Dear Editor,

I have read the new version of the manuscript NCOMMS-19-3995858A by P. Michel et al. I think the authors did a very good job and provided detailed answers, and updated accordingly the manuscript. I think the paper is much clearer now, in particular the have disambiguated "prolate" and "top-shape" meanings. Also they have redone some figures following my comments. In this conditions , in my opinion, this paper is suitable for publication.

Best regards

Sebastien Charnoz

Reviewer #2 (Remarks to the Author):

This reviewer thanks the authors for their reply and detailed explanation to the requested revision. Most of their arguments are at least as valid as the concerns raised in former review. In this sense, even if not all authors explanations are fully convincing, they look reasonable enough to support most of their conclusions and to be published.

Still, one issue deserves attention and requires further comment in the manuscript. Simulations show multiple rubble-pile formation as collections of several tens of equal size spheres into somewhat oblate shapes. This reviewer wonders: (1) Does considering SPH fragments as all equal size (and mass?) affect the final shape of the rubble-piles? (2) How does the assumption of having spherical ragments affect the final shape of such rubble-piles?

Ferrari et al. (MNRAS, 2020) and Campo Bagatin et al. (Icarus, 2020) have recently shown (right after the authors' manuscript submission) that gravitational collapse of non-spherical fragments influence the way gravitational collapse takes place and may explain most asteroids shapes.

This reviewer believes that -in order for the manuscript to be fully acceptable- it is necessary to fairly warn the reader on this issue. Therefore it is necessary to honestly state the oversimplification of the authors' re-accretion model making reference to the mentioned papers.

<u>Answers to Reviewers</u>

We thank the reviewers for their further comments and suggestions.

First, for clarity, to include some aspects of our rebuttal to Reviewer2 to our manuscript, we have bolstered our discussion regarding the contribution of YORP to the formation of top-shapes. We include the following paragraph in our Results section under the heading *Diversity of rubble-pile shapes*:

After the formation of an oblate spheroid, it should not take much time (order of 10^4 to10^6 years for kilometer-sized asteroids [3,23]) for post-processes such as YORP to lead to top shapes such as those currently observed. Dynamical studies of the spins of main belt asteroids show that YORP evolution is required to explain their distribution [24]. Measurements of Bennu's YORP acceleration show that its spin-rate doubling period is roughly 1.5 Myr at its current orbit [25]. In the inner main belt, this timescale would be ~ 6 Myr. Observations of Ryugu and Bennu require a model that forms a top shape rapidly such that subsequent impact cratering can form large craters that overlay the equatorial ridge [26,5]. The YORP-timescale provides ample time for the transformation of an oblate spheroid to a top-shape before the formation of the largest craters. For example, Bennu's largest equatorial craters (~100 m diameter) may require 0.1 to 1 Gyr residence time in the main belt to form [5]. In that scenario, large-scale YORP deformation following gravitational reaccumulation would be plausible.

Below, we respond to the final comments from reviewer 2.

<u>Reviewer 2.</u>
Still, one issue deserves attention and requires further comment in the manuscript. Simulations show multiple rubble-pile formation as collections of several tens of equal size spheres into somewhat oblate shapes. This reviewer wonders:
(1) Does considering SPH fragments as all equal size (and mass?) affect the final shape of the rubble-piles?
(2) How does the assumption of having spherical fragments affect the final shape of such rubble-piles?
Ferrari et al. (MNRAS, 2020) and Campo Bagatin et al. (Icarus, 2020) have recently shown (right after the authors' manuscript submission) that gravitational collapse of non-spherical fragments influence the way gravitational collapse takes place and may explain most asteroids shapes.
This reviewer believes that -in order for the manuscript to be fully acceptable- it is necessary to fairly warn the reader on this issue. Therefore, it is necessary to honestly state the oversimplification of the authors' re-accretion model making reference to the mentioned papers.

We thank the reviewer for pointing out this important caveat to our study. The combined effects of spherical particles and a mono-disperse size distribution plays an important role in the

collisional and gravitational dynamics of rubble piles. We have limited our study to the simple case of spherical particles with a mono-disperse size distribution in order to perform the first assessment of the gravitational reaccumulation of the smallest family members following a catastrophic disruption, and to study their thermal histories. Future work will include the important consideration of a realistic size distribution, such as one described by a power-law, and particles with non-spherical shapes.

However, we will note that, although the particles simulated here are spherical, we model inter-particle frictional forces (static, rolling, twisting) that mimic the complex behavior of non-spherical grains.

We attempt to address the reviewer's point by including the following discussion in the Methods section of our manuscript, where we describe how we model the gravitational reaccumulation phase:

Our study does not capture two considerations that may contribute second-order effects that influence the final shape of a reaccumulated remnant. The first is our simplified assumption of a monodisperse size distribution. Work by refs [22, 29, 58] have shown that a non-uniform size distribution can influence the effective internal angle of friction of a rubble pile. Those authors investigated the influence of a size distribution on the critical spin period of a rubble pile before rotational disruption; nevertheless, we surmise that this could play a role in the reaccumulation process. The second simplifying assumption in our simulations is the use of spherical particles. Recent work by [59, 60] has shown that the gravitational collapse of non-spherical fragments may influence the gravitational reaccumulation process, leading to varied final shapes.

However, our initial conditions start from the actual dynamical configuration of fragments produced by the impact phase, instead of a global collapse of a cloud of fragments, as considered in [59, 60]. We show that the reaccumulation phase does not necessarily involve a single collapse for which fragment shapes can play a role in the final outcome, but rather the interactions between precursor aggregates which gravitationally reaccumulate to form the final rubble pile. Although the shape of individual components may still have a role in this case, our modeling accounts for the various kinds of frictional forces between spherical components during precursor aggregate collisional interactions, which dominate in the final phases of a reaccumulation event.

REVIEWERS' COMMENTS:

Reviewer #2 (Remarks to the Author):

This reviewer thanks the authors for including last suggestion. In my opinion the paper may be ready for publication.
